# Degradation of textile dyes using *Biancaea sappan* extract coated zinc nanoparticle

**DOI:** 10.1038/s41598-025-29477-2

**Published:** 2025-11-28

**Authors:** Rao R. Dhanya, P. A. Aathira, K. Ashtamy Krishna, Sanjeev K. Ganesh, Sreelakshmi R. Nair, V. Mohanasrinivasan, C. Subathra Devi

**Affiliations:** Department of Biotechnology, School of Bio Sciences and Technology, Vellore Institute of Technology, Vellore, 632 014 Tamil Nadu India

**Keywords:** Biancaea sappan, ZnO, Nanoparticles, Dye pollution, Antibacterial, Biochemistry, Biotechnology, Chemistry, Environmental sciences, Materials science, Microbiology, Nanoscience and technology, Plant sciences

## Abstract

This study presents a green and efficient method for synthesizing zinc oxide (ZnO) nanoparticles using *Biancaea sappan* leaf extract, marking the first report of this plant’s application in nanoparticle fabrication. The extract coated ZnO nanoparticles (BSE-ZnONPs) displayed enhanced multifunctional bioactivity, showing strong antibacterial inhibition against *Staphylococcus sp.* (45 mm), high anti-inflammatory activity (95.57%), and notable antioxidant potential (78.65% inhibition at 500 µg/mL). Under optimized conditions (200 mg/L, pH 1, 7.5 min ultrasonication), the nanocomposite achieved 98.88% dye degradation efficiency, surpassing similar green-synthesized ZnO systems reported previously (< 95%). The BSE-ZnONPs maintained over 84% efficiency for two reuse cycles and retained moderate activity (42.7%) in the third cycle, confirming good recyclability. Cytotoxicity studies revealed high cell viability in L929 cells (IC₅₀ = 151.48 µg/mL), and in vivo assays using *Oreochromis niloticus* demonstrated significant mitigation of dye-induced histopathological damage. These results highlight the superior catalytic and biocompatible performance of BSE-ZnONPs and their potential as a sustainable nanotechnological solution for textile wastewater treatment and aquatic ecosystem protection.

## Introduction

*Biancaea sappan*, a plant belonging to the Fabaceae family, was renowned for its medicinal properties. Its heartwood yielded a red dye that had been used in Thai traditional medicine to treat various ailments. The plant contained several bioactive phenolic compounds, with brazilin and brazilein being particularly notable for their diverse therapeutic effects, including anti-inflammatory and antioxidant properties^1,2^. Nanotechnology had advanced rapidly, offering significant potential for various innovations^3^. While physical and chemical methods for nanoparticle synthesis existed, they often had drawbacks such as high costs and environmental concerns^4–6^. Biological synthesis using plants, particularly medicinal plants, offered an eco-friendlier and more cost-effective alternative^7^. Zinc oxide (ZnO) nanostructures were favour for their affordability, ease of use, and applicability in various fields. They exhibits beneficial properties such as chemical stability, photocatalytic activity, and biocompatibility^8–10^. Alongside conventional approaches, emerging strategies in nanoconfined catalyst engineering and advanced phytochemical profiling of plant extracts highlight new directions for designing bio-inspired functional nanoparticles^11,12^.

Many recent studies have utilized radiation-driven oxidative techniques, such as UV–vis and gamma irradiation, for the degradation of textile and azo dyes in wastewater. These approaches have demonstrated effective decolourization and pollutant breakdown through the generation of reactive oxidative species. However, such methods often require high energy input, specialized radiation sources, and controlled reaction conditions, which may limit their large-scale applicability. In contrast, the present study employs a sonocatalytic mechanism using ultrasonication (20 kHz) combined with a green, plant-extract-mediated ZnO nanoparticle system that operates efficiently under ambient conditions, offering a more sustainable and energy-efficient alternative for dye degradation^13,14^.

The textile industry, one of the world’s oldest and largest, posed significant environmental challenges due to its effluents. These effluents, rich in organic dyes, could severely impact aquatic ecosystems^15^. Many of these dyes were toxic, carcinogenic, and resistant to degradation^16,17^. Fish, particularly tilapia played a crucial role both as a food source and as bioindicators of water pollution^18^. Exposure to pollutants could cause various physiological and biochemical changes in fish^19,20^. Green synthesized ZnO nanoparticles showed promise for treating textile industry wastewater^21,22^. Adsorption using these nanoparticles was widely used due to its economic viability and performance^23,24^. Despite extensive research on the effects of industrial effluents on fish there was still a lack of data on the specific impact of textile dyes on fish. Thus, integrating nanoparticle-based remediation with ecotoxicological assessment provides a holistic understanding of how green-synthesized ZnO nanoparticles can simultaneously degrade textile dyes and reduce their toxic effects on aquatic organisms such as tilapia.

Despite several reports on green-synthesized ZnO nanoparticles using plant extracts such as *Euphorbia hirta* and *Scoparia dulcis*^25,26^, there remains a need to explore phytochemical sources with higher reductive potential and diverse secondary metabolites for improved nanoparticle functionality. *Biancaea sappan*, a medicinal plant rich in phenolic compounds such as brazilin, brazilein, and conjugated linoleic acids, exhibits exceptional antioxidant and anti-inflammatory properties^27,28^. These biomolecules are known to act as natural reducing and capping agents, enhancing nanoparticle stability and bioactivity. Unlike *Euphorbia hirta* and *Scoparia dulcis*, which primarily contain flavonoids and terpenoids, *B. sappan* possesses naphthoquinones and phenolic lignans that contribute to higher electron-donating capacity and stronger radical scavenging potential. Therefore, *B. sappan* was selected as a novel and potent biogenic source for ZnO nanoparticle synthesis aimed at enhancing dye degradation efficiency and biocompatibility.

This study aimed to address this gap by synthesizing *Biancaea sappan* leaf extract–coated zinc oxide nanoparticles for the degradation of textile dyes and evaluating their effects on tilapia, focusing on behavioral, morphological, and histopathological parameters. In addition, it explored the potential of *Biancaea sappan* leaf extract in the green synthesis of ZnO nanoparticles and examined their antioxidant, antibacterial, and anti-inflammatory properties^29^. Therefore, the present study specifically aims to (i) synthesize *Biancaea sappan* leaf extract–coated ZnO nanoparticles via a green route, (ii) characterize their physicochemical and biological properties, (iii) evaluate their efficiency in degrading multiple textile dyes, and (iv) assess their cytotoxic and ecotoxic effects using in-vitro (L929 cells) and in-vivo (*Nile tilapia*) models. It is hypothesized that the plant extract–coated ZnO nanoparticles will exhibit enhanced dye degradation efficiency and reduced toxicity compared to uncoated nanoparticles or untreated dyes, thereby providing a sustainable and eco-friendly approach for industrial effluent remediation. This work is novel in integrating *Biancaea sappan* mediated green synthesis of ZnO nanoparticles with sonocatalytic dye degradation and comprehensive ecotoxicological evaluation, demonstrating a unified, sustainable, and biocompatible strategy for mitigating the environmental impact of textile dye pollution.

## Materials and methods

### Extraction and characterization of *Biancaea sappan* extract

*Biancaea sappan* leaves were collected from Calicut, Kerala during monsoon time. After being cleaned and shade dried, ground leaves of about 20 g were boiled for 15 min in 100 mL of distilled water. The extract was cooled and filtered then mixed with 50 mL of ethanol. The mixture was centrifuged at 10,000 rpm for 10 min, and the process was repeated twice. The supernatant was collected and stored at 4 °C. GC-MS analysis was conducted to characterize the bioactive compounds present in the ethanolic extract^30^.

### Synthesis of ZnO nanoparticle using plant extract and characterization

ZnO nanoparticles were synthesized by combining100 mL of the plant extract and 6 g of zinc nitrate. A 0.2 M solution of zinc nitrate hexahydrate (Zn(NO₃)₂·6 H₂O) was prepared, and 6 g (corresponding to 0.02 mol) was used for the reaction. A magnetic stirrer at 60 °C was used to homogenize the mixture for 2 h. The solution was centrifuged at 10,000 rpm for 10 min after cooling to 25 °C. The obtained pellets were washed three times with distilled water and dried in a hot air oven at 90 °C. Using a mortar and pestle the dried extract was then ground into a fine powder. ZnO nanoparticles were synthesized when the powdered mixture was calcinated at 500 °C for 2 h. The yield of ZnO nanoparticles obtained after calcination was approximately 78% based on the initial zinc nitrate content.

Multiple analytical techniques were used for the characterization of synthesized nanoparticles.The absorbance of the nanoparticles was analyzed using a UV spectrometer and crystalline structure was determined through X-ray diffraction (XRD) analysis using a Bruker AXS D8 Advance diffractometer with Cu K-alpha radiation (wavelength 1.5402 Å). To identify functional groups in the nanoparticles, Fourier-transform infrared spectroscopy (FTIR) was performed utilizing a Thermo Fisher Scientific Nicolet iS50 FT-IR spectrophotometer. The FTIR spectra were analyzed within the range of 400–4000 cm^−1^, as described by Faisal et al. (2021)^31^. To examine the size and morphology of the ZnO nanoparticles a Field Emission Scanning Electron Microscope (FESEM) was employed. The elemental composition of the ZnO nanoparticles was confirmed through Energy Dispersive X-ray (EDX) mapping^32^.

### Synthesis of plant extract-loaded ZnO nanocomposite

A chitosan solution was prepared by dissolving chitosan in 0.3% glacial acetic acid at a 1:1 ratio. To synthesize chitosan-coated ZnO nanoparticles (NPs), an equal amount of ZnO NPs was added to the chitosan solution and thoroughly mixed using a magnetic stirrer for 3 h. The homogeneous mixture obtained was centrifuged at 5000 rpm for 20 min, and the pellet was dried to obtain the chitosan-coated ZnO NPs composite. Plant extract (50 mg) was dissolved in 10 mL of ethanol. To this solution, 50 mg of the previously prepared chitosan-coated ZnO NPs composite was added and vortexed for 24 h^33^. The mixture was then centrifuged, and the resulting pellet was dried to yield the *Biancaea sappan* extract-coated chitosan-ZnO NPs composite (BZnO-NPs).

### Analysis of antimicrobial, antioxidant, and anti-inflammatory activities of BSE-ZnO NPs

#### Antimicrobial assay

The antibacterial efficacy of ZnONPs, *Biancaea sappan* leaf extract and BSE-ZnO NPs evaluated against *Klebsiella* *sp*. *Pseudomonas* *sp*. *Staphylococcus* *sp*. and *E. coli*. The in vitro antibacterial activity was assessed using the well diffusion method on Muller Hinton Agar (MHA)^34^. Fresh pathogens were inoculated on MHA agar plates, and 100 µg/mL of ZnONPs, *Biancaea sappan* leaf extract and BSE-ZnO NPs, were added to separate wells. Ciprofloxacin and DMSO were used as positive and negative controls, respectively. After a 24 h incubation at 37 °C, the zone of inhibition was measured^31^.

#### Antioxidant activity

Ascorbic acid, BSE-ZnO NPs, ZnONPs, and *Biancaea sappan* leaf extract were produced at concentartions ranging from 100 to 500 µg/mL. Methanol was added to each sample after it had been combined with 2.5 mL of 0.1 mM DPPH solution to reach a final volume of 3 mL. The control was a pure DPPH solution, and the standard was ascorbic acid. A UV spectrophotometer was used to detect the absorbance at 517 nm following a 20 min dark incubation period^35^. The following formula was used to determine the percentage of inhibition:

$$\% {\rm Inhibition} = (A_{0}-A_{1})/A_{0} \times 100.$$

where: A_0_ = Absorbance of control, A_1_ = Absorbance of test sample.

#### Anti-inflammatory activity

The in-vitro anti-inflammatory assay was performed using human blood obtained from healthy, consenting adult volunteers who had abstained from NSAIDs for two weeks prior to sampling. The procedure was approved by the Institutional Ethics Committee of Vellore Institute of Technology (Approval No: VIT/IECH/2025/16 IECH/8 February 2025/4). An equal volume of Alsever’s solution was then mixed with the collected blood, followed by centrifugation and washing to prepare a 10% cell suspension. Various concentrations (100–500 µg/mL) of ZnO NPs, BSE-ZnO NPs, and Biancaea sappan leaf extract in glacial acetic acid were combined with phosphate buffer, hyposaline, and the cell suspension. After 30 min of incubation at 37 °C, the mixtures were centrifuged, and absorbance was measured at 560 nm^36^. The percentage of anti-inflammatory activity was calculated using the formula:

$$\% {\text{Anti-inflammatory Activity}}=(A_{0}-A_{1})/A_{0}\times 100.$$

where: A_0_ = Absorbance of control A_1_ = Absorbance of sample.

#### Dye degradation analysis

The dye degradation bioactivity of *Biancaea s*appan extract (BSE)-coated ZnO nanoparticles (NPs) was evaluated using six different dyes: Crystal violet (CV), Congo red (CR), methyl orange (MO), methyl red (MR), bromothymol blue (BTB), and Coomassie brilliant blue (CBB). Each dye solution (0.25 ppm) was prepared in 50 mL of distilled water. BSE-ZnO NPs (150 mg) were added to each dye solution and homogenized for 3 h using a magnetic stirrer at 485 rpm. Initial and final optical density (OD) measurements were recorded at specific wavelengths for each dye: CR (495 nm), MO (415 nm), CBB (565 nm), BTB (547 nm), and CV (590 nm)^29^. The dye degradation was further analyzed using FTIR spectroscopy with an Agilent Cary 630 FTIR spectrophotometer.

$$\eta = (C_{0}-C)/C_{0}\times 100$$

where: η = Dye degradation efficiency (%) C₀ = Initial concentration of the dye C = Final concentration of the dye.

### Optimization of BSE-ZnO NPs to enhanced dye degradation

The efficiency of dye degradation by BSE-ZnO NPs was enhanced by varying several parameters. These included the dosage of the nanocomposite (20, 80, 150, and 300 mg), agitation methods (static, magnetic stirring at 480 rpm, and ultrasonication at 20 kHz), reaction time (10, 20, and 30 min), and dye concentration (0.35, 0.45, 0.55, 0.65, and 0.75 mL of dye solution.In 100 mL conical flasks, a mixture of all five dyes (0.25 ppm each) was made in 50 mL of distilled water, and the efficiency of dye degradation was examined under the specified circumstances^37^. The dye degradation efficiency was monitored by measuring the OD using a UV-Vis spectrophotometer, recording both initial and final readings for each condition tested.

$$\eta = (C_{0}-C)/C_{0}\times 100$$

where: η = Dye degradation efficiency (%) C₀ = Initial concentration of the dye C = Final concentration of the dye.

### Statistical optimization of BSE-ZnONPs for dye degradation using response surface methodology (RSM)

The effects of three independent variables on a combination of dyes were examined using a Box-Behnken statistical design: Time (A, min), BSE-ZnONPs (B, mg/L), and pH (C). The experimental design comprised 17 runs with varying combinations of these factors, as detailed in Table 1. In each experiment, five dyes were introduced to 100 mL conical flasks with 50 mL of distilled water at an equal concentration of 0.25 ppm each. Each run’s agitation duration, pH, and BSE-ZnONP composite concentration were modified in accordance with the design specifications.All experiments were carried out in triplicate to ensure reliability and Response Surface Methodology (RSM) analysis was performed using the average results. Analysis of Variance (ANOVA) was used to statistically validate the dependent variable, dye degradation efficiency (designated as response 1). A UV-Vis spectrophotometer was used to measure the absorbance of dye solution at 582 nm after each experimental run in order to assess the degree of dye degradation^38^.

### Analysing the reusability of BSE-ZnONP

The reusability of BSE-ZnONPs was evaluated through multiple cycles of dye mixture degradation. In a conical flask containing 50 mL of distilled water was used to prepare a solution of all five dyes at a concentration of 0.25 ppm. To this solution, 200 mg of BSE-ZnONPs were added and subjected to ultrasonication at 20 kHz for 7.5 min at pH 1. After completing the first degradation cycle, the BSE-ZnONPs were recovered by filtration using Whatman No. 1 filter paper. The filtered nanoparticles were then washed with ethanol and distilled water before being dried in a hot air oven at 60 °C. These recovered nanoparticles were subsequently used for two additional degradation cycles. The dye degradation efficiency was calculated for each of the three cycles to assess the reusability of the BSE-ZnONPs.

### In-vitro toxicity analysis of BSE- ZnON using microscopic analysis and MTT assay

Dulbecco’s Modified Eagle’s Medium (DMEM), which was acquired from Sigma Aldrich in the United States, was used to sustain L929 mouse fibroblast cells that were acquired from NCCS in Pune, India. The cells were cultured in 25 cm³ flasks with 10% FBS, L-glutamine, antibiotics, and sodium bicarbonate (Merck, Germany). The incubation was carried out in a humidified environment at 37 °C with 5% CO₂ (NBS Eppendorf, Germany). The MTT assay technique and direct inspection under an inverted phase-contrast microscope were used to assess cell viability. A confluent cell monolayer that was two days old was suspended in 10% growth media after being trypsinized. A 96-well plate was seeded with 100 µL of a cell suspension (5 × 10³ cells/well) and allowed to incubate. After dissolving 1 mg of the test substance in 1 mL of 0.1% DMSO, the mixture was filter-sterilized. Serial dilutions of the chemical in DMEM (100 µg, 50 µg, 25 µg, 12.5 µg, and 6.25 µg in 500 µL) were used in place of the growth medium after a 24 h period. The wells were then incubated after 100 µL samples of each concentration were added in triplicate. For comparison, untreated controls were kept. After a day, cytotoxicity was evaluated by direct microscopic inspection with an Optika Pro5 CCD camera attached to an Olympus CKX41 microscope. As markers of cytotoxicity, morphological alterations like vacuolization, cytoplasmic granulation, and cell shrinkage were noted.For the MTT assay, 15 mg of MTT (Sigma, M-5655) was reconstituted in 3 mL of PBS and filter-sterilized. After the test material was incubated for 24 h, 30 µL of MTT solution was added to each well and incubated for 4 h. After removing the supernatant, 100 µL of DMSO was added to dissolve the formazan crystals. To ensure homogeneity, the wells were pipetted carefully^39^.

### In-vivo toxicity analysis of BSE- ZnON on *Oreochromis niloticus* (Nile tilapia)

The experimental study involved Tilapia fish obtained from a farm in Walajapet, Tamil Nadu, India. A total of 100 fish, with an average weight of 40.2 ± 1.42 g and a length of 9 ± 0.4 cm, were transported to the laboratory. To ensure their health, the fish were treated with 0.4% formalin for 15 min, followed by a 1 h exposure to 1 mg/L potassium permanganate to eliminate parasites and infections. After that, the fish were acclimated for ten days in glass aquariums with controlled conditions, such as a pH of 7, temperatures between 25 and 28 °C, and sufficient aeration. They were given Tokyo artificial fish feed, and daily 75% water changes were done to preserve the water quality. Four groups of twelve fish each were divided for the experiment: a control group, a drug control group treated with extract-coated ZnO-NPs, a dye-exposed group, and a treated group in which extract-coated ZnO NPs and ultrasonication were used to break down the dye. The study was conducted over 96 h, during which behavioral and morphological changes were closely observed and recorded. At the end of the study, the fish were dissected to obtain intestine, gill, and muscle samples for histopathological analysis^40,41^. These tissue specimens were preserved in formalin, processed using paraffin embedding techniques, sectioned, and stained with Hematoxylin and Eosin for detailed examination.

### Statistical analysis

All statistical analyses were conducted in triplicate and results are expressed as mean ± standard error (SE). Data were analyzed using Origin 8.2 and GraphPad Prism version 3.04.

## Results

### GCMS analysis of *Biancaea Sappan* extract

The GC-MS analysis of the ethanol extract from *Biancaea sappan* leaves identified 15 bioactive compounds, including fatty acids such as n-hexadecanoic acid, oleic acid 9,12-octadecadienoic acid (Z, Z), 10(E),12(Z) conjugated linoleic acid, 6-octadecanoic acid, and 9-octadecanoic acid (E), as well as naphthalene, a compound known for its anti-cancer properties. A bioactive compound 9(E),11(E)-conjugated linoleic acid was detected, exhibiting dual anti-cancer and anti-inflammatory activities.

### Characterization of ZnO nanoparticle

**Fig. 1:**
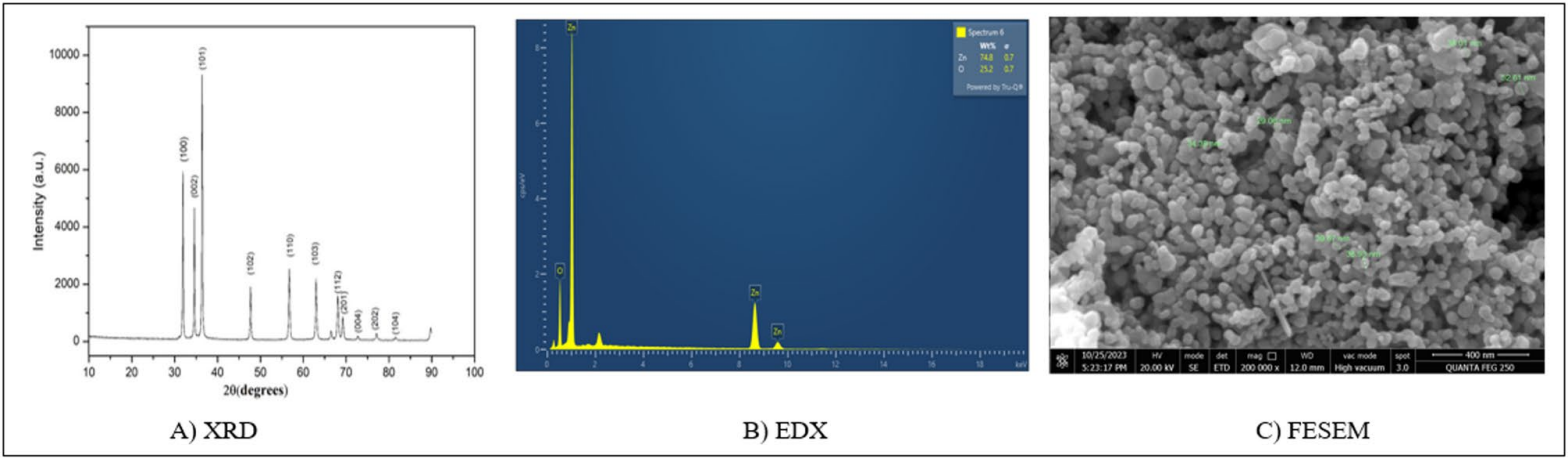
(**A**) XRD image of synthesized ZnO nanoparticles, (**B**) EDX mapping showing the presence of Zn and O elements, (**C**) FESEM image visualizing the spherical morphology of the ZnO nanoparticles.

The synthesized ZnO nanoparticles appeared as a 5gn -colored fine powder. UV-visible spectroscopy, which showed a distinctive absorption peak in the 360–380 nm range, verified the production of ZnO nanoparticles. The crystalline structure of the nanoparticles was further confirmed by X-ray diffraction (XRD) analysis, which showed distinct peaks observed at 47.5°, 56.6°, 62.8°, 66.3°, 67.9°, 69.1°, 72.5°, 76.9°, and 81.3° (Fig. 1A). These peaks corresponded to the standard reference (JCPDS card number 00–036-1451), confirming the successful synthesis of ZnO nanoparticles. The elemental composition of the nanoparticles was verified by energy-dispersive X-ray spectroscopy (EDX) mapping, which showed the presence of oxygen and zinc. (Fig. 1B). The size and shape of the nanoparticles were analyzed using Field Emission Scanning Electron Microscopy (FESEM). (Fig. 1C). The results indicated that the particles were spherical in shape, heavily clustered, and uniformly distributed, with a size range of 29.06 to 52.61 nm.

### FTIR analysis of Biancaea Sappan extract loading on Chitosan coated ZnO nanoparticles

FTIR analysis confirmed the successful loading of Biancaea sappan extract onto chitosan-coated ZnO nanoparticles. The spectrum of B. sappan extract showed peaks at 3257.869 cm^−1^ (N–H), 2975.766 cm^−1^ (N–H), 1376.735 cm^−1^ (S=O), 1122.407 cm^−1^ (S=O), and 1270.756 cm^−1^ (C–N). Chitosan exhibited peaks at 1058.034 cm^−1^ (C–O), 1332.274 cm^−1^ (S=O), 1379.731 cm^−1^ (O–H), and 1027.563 cm^−1^ (C–O), while ZnO nanoparticles displayed peaks at 1025.384 cm^−1^ (C–O), 1522.741 cm^−1^ (N–O), 1718.368 cm^−1^ (C=O), and O-H peaks at 3576.131 cm^−1^, 3631.369 cm^−1^, and 3713.968 cm^−1^. The composite spectrum revealed peaks at 1756.938 cm^−1^ (C=O), 2930.031 cm^−1^ (C–H), 3245.239 cm^−1^ (N–H), 1021.189 cm^−1^ (C–N), 1247.975 cm^−1^ (C–O), 1149.683 cm^−1^ (C–O), 1117.538 cm^−1^ (S=O), 1340.531 cm^−1^ (S=O), 1063.486 cm^−1^ (C–O), and 1399.222 cm^−1^ (O–H). The presence of characteristic peaks from all three components confirmed the successful loading of the plant extract on chitosan-ZnO nanoparticle composite.

### Evaluation of bioactive properties: BSE-ZnONP antimicrobial, antioxidant, and anti-inflammatory efficacy

**Fig. 2:**
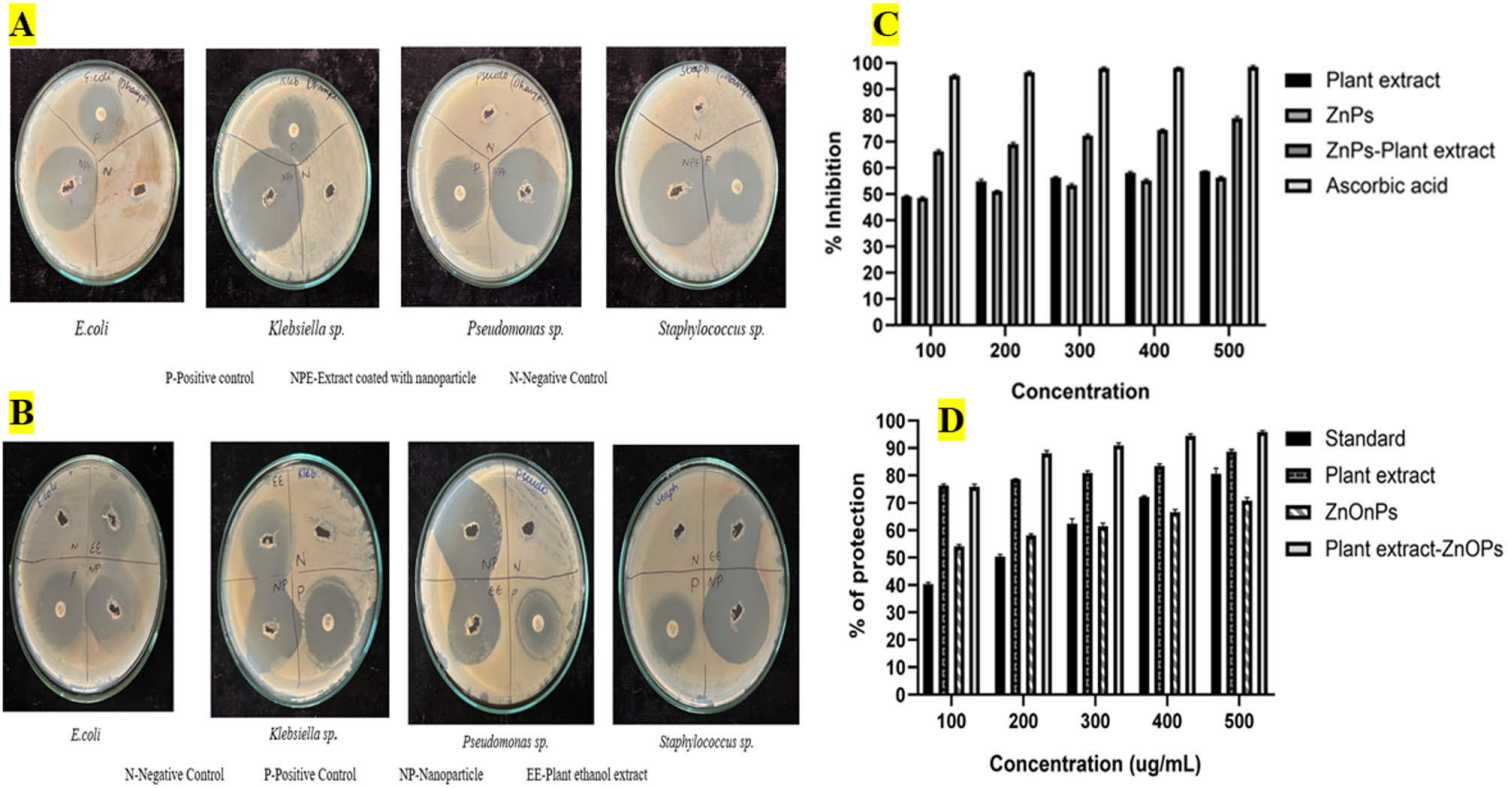
(**A**) Antimicrobial activity of extract coated NPs against different pathogens. (**B**)Antimicrobial activity of *Biancaea sappan extract* and ZnO NPs against different pathogens (**C**) Antioxidant activity of plant extract, ZnNPs, ZnNPs-plant extract and ascorbic acid, (**D**) Anti-inflammatory activity of standard, plant extract, ZnONPs, and ZnONPs-plant extract.

The antibacterial efficacy of the *Biancaea sappan extract* (BSE), ZnO-NPs, and extract-coated NPs was evaluated against various bacterial strains. The plant extract exhibited inhibition zones of 25 mm for *Staphylococcus sp.* 26 mm for *E. coli*, 33 mm for *Klebsiella sp.* and 34 mm for *Pseudomonas sp.*, with the highest activity observed against *Klebsiella*  *sp*. and *Pseudomonas sp.* ZnO NPs were observed to had larger inhibition zones of 40 mm for *Staphylococcus sp*., 31 mm for *E. coli*, 37 mm for *Klebsiella sp.* and 36 mm for *Pseudomonas sp*., showing peak effectiveness against *Staphylococcus* *sp*. and *Klebsiella sp.* (Fig. 2)

The extract-coated NPs exhibited the most significant antibacterial activity, with inhibition zones of 45 mm for *Staphylococcus sp*., 36 mm for *E. coli*, 44 mm for *Klebsiella sp*., and 40 mm for *Pseudomonas sp*., with the highest efficacy noted against *Staphylococcus* and *Klebsiella sp.* (Fig. 2). Overall, the extract-coated NPs exhibited the strongest antibacterial effect, particularly against *Staphylococcus sp.* (45 mm), while the plant extract alone showed the least activity against the same bacterium (25 mm) (Fig. 2). Ciprofloxacin, used as a positive control, displayed maximum effectiveness against *Pseudomonas sp.* (30 mm) and *Klebsiella sp.* (33 mm).

For antioxidant activity, the inhibition percentage increased with concentration across all samples. At 500 µg/mL, the highest inhibition (78.65%) was observed for the extract-coated NPs and the lowest inhibition (55.88%) was observed with ZnO NPs. The plant extract alone demonstrated intermediate activity (58.04%). Similarly, anti-inflammatory activity, measured as a percentage, increased with concentration for all samples. At 500 µg/mL, the highest anti-inflammatory activity (95.57%) was observed for the extract-coated NPs, while the lowest activity (71.03%) was observed for ZnO NPs. The plant extract was observed to have intermediate activity at 88.88% (Fig. 2). In all three evaluations, the extract-coated NPs demonstrated significantly higher activity than the other samples, especially at higher concentrations. In contrast, ZnO NPs alone consistently showed the lowest effectiveness in antibacterial, antioxidant, and anti-inflammatory activities.

### Dye degradation analysis and optimization using BSE-ZnONP

The degradation efficiency of Congo Red (CR), Crystal Violet (CV), Bromothymol Blue (BTB), Coomassie Brilliant Blue (CBB), and Methyl Orange (MO) was evaluated using extract-coated ZnO nanoparticles. Optical density (O.D) measurements at 0 h and after 3 h revealed that CBB exhibited the highest degradation efficiency (66.9%), while MO showed the lowest (10.33%). For CR, the absorbance peak at 520 nm decreased significantly, achieving 64.4% degradation. FTIR analysis confirmed this, as key peaks in the control (e.g., 1273.445 cm^−1^, 1527.350 cm^−1^) disappeared, and new peaks (e.g., 1076.136 cm^−1^, 1636.735 cm^−1^) emerged, indicating structural changes. Similarly, CV degradation at 590 nm reached 42.2%, with FTIR showing the absence of control peaks (e.g., 1153.413 cm^−1^, 1225.776 cm^−1^) and the appearance of new peaks (e.g., 2850.287 cm^−1^, 3286.136 cm^−1^). BTB degradation at 435 nm showed 41.2% efficiency, with FTIR confirming the disappearance of control peaks (e.g., 1606.439 cm^−1^, 1154.407 cm^−1^) and the formation of new peaks (e.g., 1628.673 cm^−1^, 1156.363 cm^−1^).

CBB degradation at 592 nm achieved the highest efficiency of 66.9%, supported by FTIR analysis, which revealed the disappearance of control peaks (e.g., 1031.440 cm^−1^, 1189.873 cm^−1^) and the appearance of new peaks (e.g., 3284.875 cm^−1^, 1238.500 cm^−1^). In contrast, MO exhibited minimal degradation, with only 10.33% efficiency at 507 nm. FTIR analysis indicated limited structural changes, as most control peaks (e.g., 1420.014 cm^−1^, 1159.833 cm^−1^) remained similar, though new peaks (e.g., 1730.090 cm^−1^, 1242.860 cm^−1^) suggested minor alterations. Efficiency of dye degradation was tabulated in Table 1. Visual observations of color changes further supported the degradation process. These findings highlight their applicability in wastewater treatment, particularly for dyes like CBB and CR, which showed higher degradation rates.

**Table 1:** Efficiency of different dye degradation by BSE-ZnONP.

Dyes used	Initial O.D values	Final O.D values (after 3 h)	Dye degradation efficiency (%)
BTB	0.916	0.538	41.2
CBB	0.877	0.290	66.9
CR	0.428	0.152	64.4
CV	0.478	0.276	42.2
MO	0.329	0.295	10.33
MR	1.508	0.910	39.65

### Optimization of dye degradation using BSE-ZnONP

The optimization studies revealed significant variations in degradation efficiency based on the tested parameters. The lowest degradation (9.17%) was observed at a nanocomposite dosage of 20 mg/mL, while the highest degradation (87.15%) was achieved at 300 mg/mL. Agitation methods also played a critical role, with static conditions yielding the least degradation (17.80%), followed by magnetic stirring (87.49%). Ultrasonication proved most effective, achieving 82.27% degradation, accompanied by a visible color change from dark blue to light. The effect of reaction time was less significant, with ultrasonication for 10 min yielding the highest degradation efficiency (87.95%), followed by 20 min (87.66%) and 30 min (86.31%). pH had a notable impact, with the highest degradation efficiency (91.66%) observed at pH 3, compared to the lowest (59.26%) at pH 9. Visual color changes were observed across all conditions, further confirming the degradation process. The optimization studies demonstrated that higher nanocomposite dosage, ultrasonication, and acidic pH conditions significantly enhance dye degradation efficiency. The application of BSE-ZnO NPs in wastewater treatment can be scaled up with the help of these findings. Preliminary optimization identified 10 min of ultrasonication as yielding high degradation efficiency (87.95%), whereas subsequent RSM analysis determined 7.5 min as the statistically optimized duration, achieving 98.88% degradation under combined optimal parameters.

### Statistical optimization of BSE-ZnONPs for dye degradation using RSM

Box-Behnken method was used to examine how time, ZnO NP composite concentration, and pH affect the degradation of a dye mixture. The experiment’s consistency was evidenced by similar results across replicate runs, with peak degradation of 95.40% observed in run 7 (Table 2). Statistical analysis revealed that a linear model best fit the data, showing the highest F value of 51.15.

The system generated a second-order polynomial equation to predict dye degradation efficiency: 82.96088 + 0.333000 A + 0.067325B − 2.05250 C.

Where A, B, and C represent time, extract concentration, and pH, respectively.

**Fig. 3:**
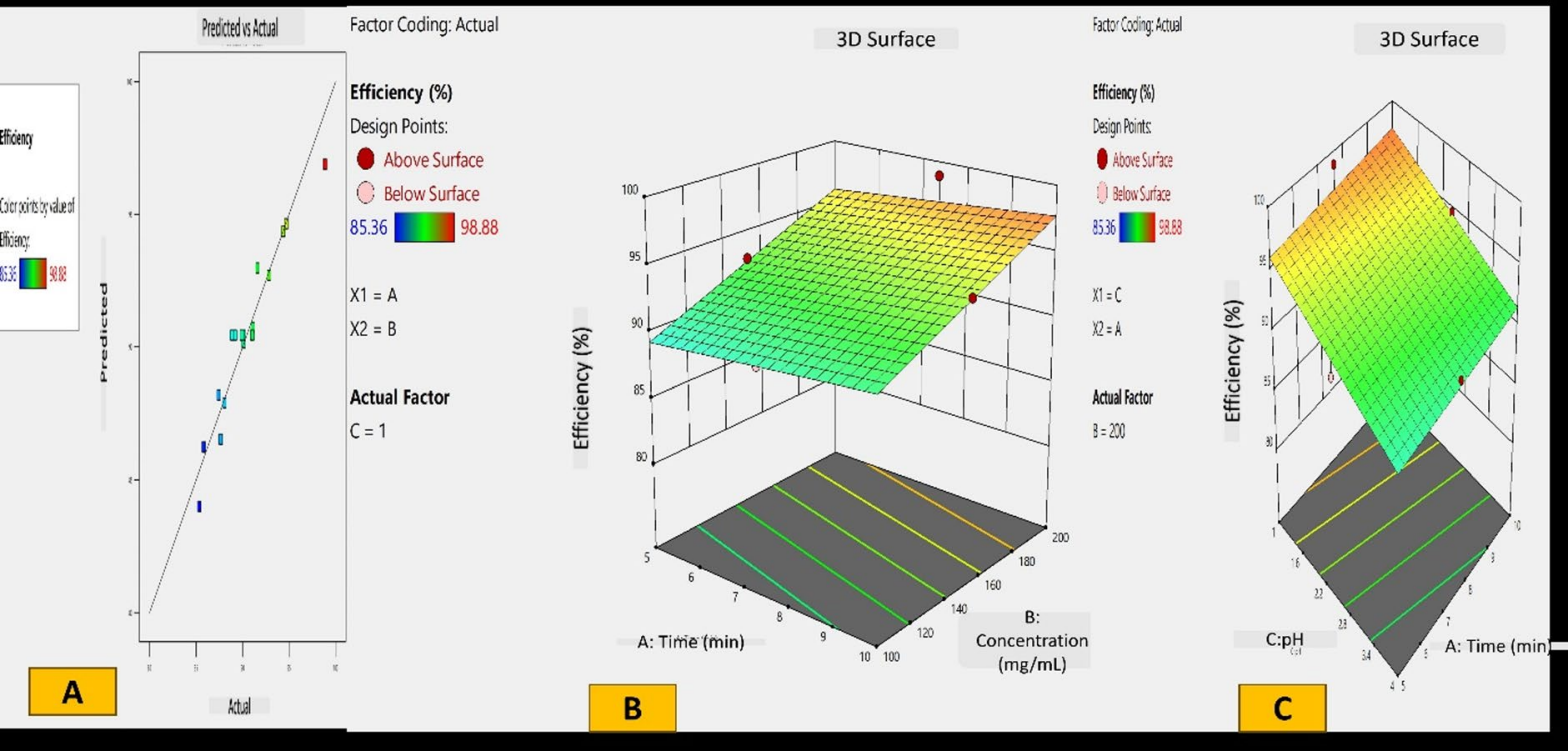
(**A**) Predicted vs. Actual relationship graph for dye degradation. 3D illustration of interaction between time and pH; (**B**); time and concentration (**C**) for dye degradation.

ANOVA results indicated high significance for the regression (*p* < 0.0001) with an R^2^ value of 0.9219. The close values of adjusted R^2^ (0.9039) and predicted R^2^ (0.8602) suggested a good model fit, explaining 99.59% of the response with minimal variability (Table 3). Comparison of F values showed that extract concentration (B) had the strongest influence on degradation efficiency, followed by pH (C), while time (A) had the least impact. The predicted vs. actual graph demonstrated strong correlation between experimental and predicted values, validating the experiment’s accuracy (Fig. 3A). 3D graphs illustrated variable interactions, with optimal conditions yielding a maximum degradation efficiency of 98.88% (Fig. 3B,C). Based on these analyses, the study determined that the optimal parameters for maximum dye degradation were 7.5 min, 200 mg/L extract concentration, and pH 1. These findings provide valuable insights into optimizing the dye degradation process using ZnO NP composites.

**Table 2:** Efficiency of dye degradation on each cycle.

Std	Run	Factor 1 A: Time (Min)	Factor 2 B: Concentration(mg/L)	Factor 3 C: pH	Efficiency %
15	1	7.5	150	2.5	90.11
8	2	10	150	4	97.45
9	3	7.5	100	1	90.12
7	4	5	150	4	87.66
1	5	5	100	2.5	85.84
16	6	7.5	150	2.5	89.21
10	7	7.5	200	1	98.99
3	8	5	200	2.5	91.61
17	9	7.5	150	2.5	89.93
14	10	7.5	150	2.5	88.89
13	11	7.5	150	2.5	91.06
11	12	7.5	100	4	85.36
4	13	10	200	2.5	94.71
2	14	10	100	2.5	88.07
5	15	5	150	1	92.84
12	16	7.5	200	4	91.12
6	17	10	150	1	94.38

**Table 3:** ANOVA table for dye degradation.

Source	Sum of Squares	Df	Mean Square	F-value	*p*-value	
**Model**	172.03	3	57.34	51.15	< 0.0001	Significant
A-Time	5.54	1	5.54	4.95	0.0445	
B-Concentration	90.65	1	90.65	80.86	< 0.0001	
C-Ph	75.83	1	75.83	67.64	< 0.0001	
**Residual**	14.57	13	1.12			
Lack of Fit	11.71	9	1.30	1.81	0.2968	Not Significant
Pure Error	2.87	4	0.7172			
**Cor Total**	186.60	16				

### Analysing the reusability of BSE-ZnONp

The reusability study revealed a gradual decline in degradation efficiency over three cycles. In the first cycle, the BSE-ZnO NPs achieved a high degradation efficiency of 90.07%. This efficiency decreased to 84.813% in the second cycle and further to 42.757% in the third cycle. Despite the observed decline, the nanoparticles retained a significant degradation capacity of approximately 40% even after three cycles of reuse. This demonstrates the potential of BSE-ZnO NPs for multiple uses in dye degradation applications, highlighting a balance between efficiency and recyclability that could be advantageous for practical wastewater treatment processes. The reusability analysis underscores the durability and potential of BSE-ZnO NPs for repeated use in dye degradation.

### In-vitro toxicity analysis of BSE- ZnON using microscopic analysis and MTT assay

**Fig. 4:**
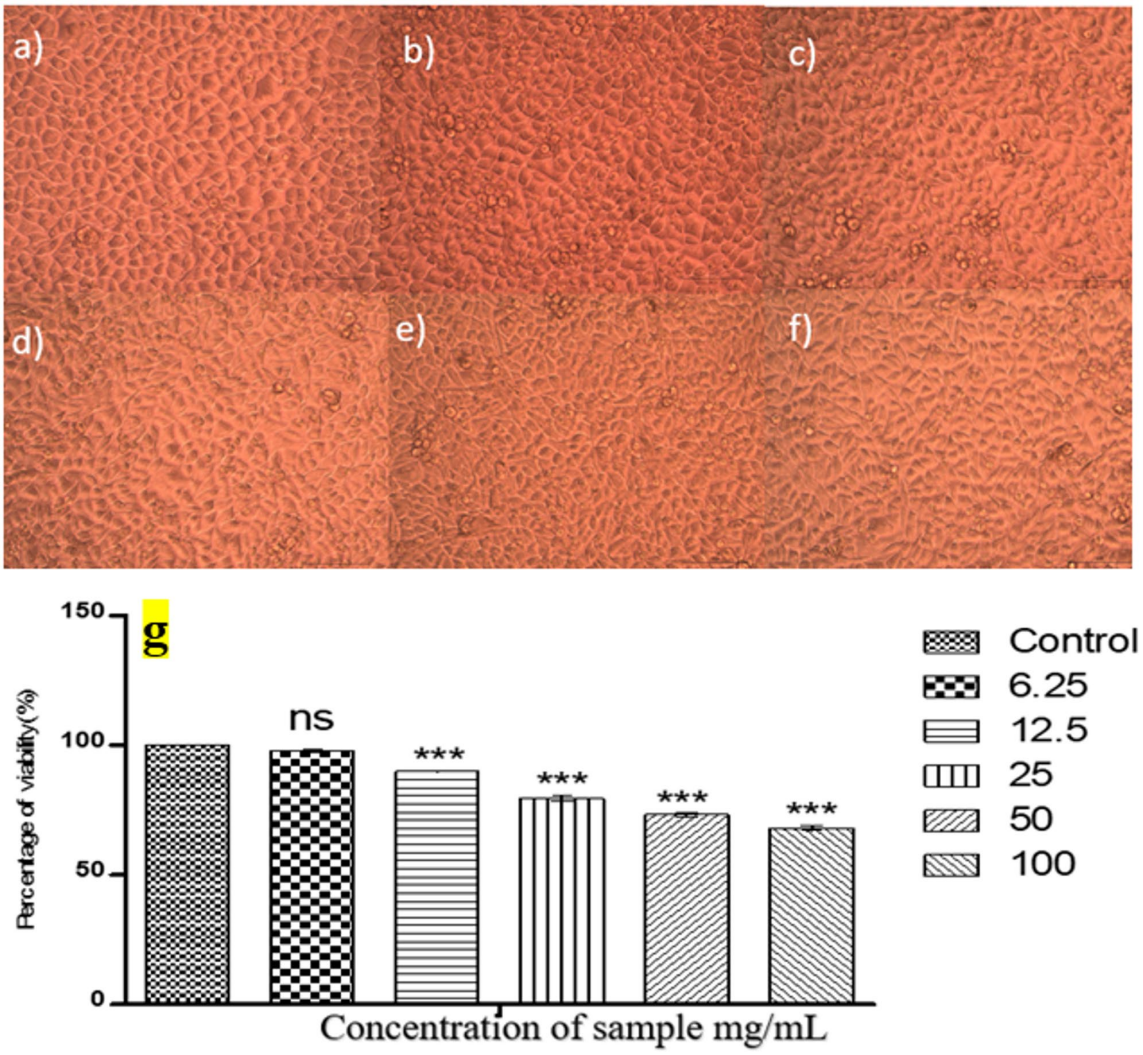
Microscopic images of the control group (**a**) and the test groups treated with varying concentration (**b**) 6.25 mg/mL, (**c**) 12.5 mg/mL, (**d**) 25 mg/mL, (**e**) 50 mg/mL and (**f**) 100 mg/mL of the BSE ZnO NPs. (**g**) Graphical representation depicting the anticancer effect of BSE coated ZnO-NP on L929 cell line by MTT assay.

The cytotoxicity of BSE-ZnONP using microscopic imaging and MTT assay was analysed in L929 cell (Fig. 4a). The microscopic analysis revealed no significant morphological changes in cells treated with various concentrations of BSE-ZnONP compared to the control group. The absence of cell rounding, shrinkage, cytoplasmic granulation, or vacuolization suggested that the nanoparticles were non-toxic at the tested concentrations. The MTT assay quantified cell viability, with the control group’s viability set at 100% for comparison. The viability of cells treated with BSE ZnO NPs ranged from 97.87% to 68.13% at concentrations ranging from 6.25 mg/mL to 100 mg/mL. The nanoparticles only showed mild cytotoxic effects at the maximum dose of 100 µg/mL. 151.4867 mg/mL was found to be the IC50 value, which further supports the nanoparticles’ cell compatibility. The qualitative visual characterization aligned with the MTT assay results, confirming the low toxicity of the nanoparticles (Fig. 4b). The BSE-ZnONP demonstrated high cell viability and minimal toxicity, with only slight effects observed at higher concentrations. This suggests their potential suitability for various biomedical applications.

### In-vivo behavioural and morphological toxicity analysis of BSE- ZnON on *Oreochromis niloticus* (Nile tilapia)

**Fig. 5:**
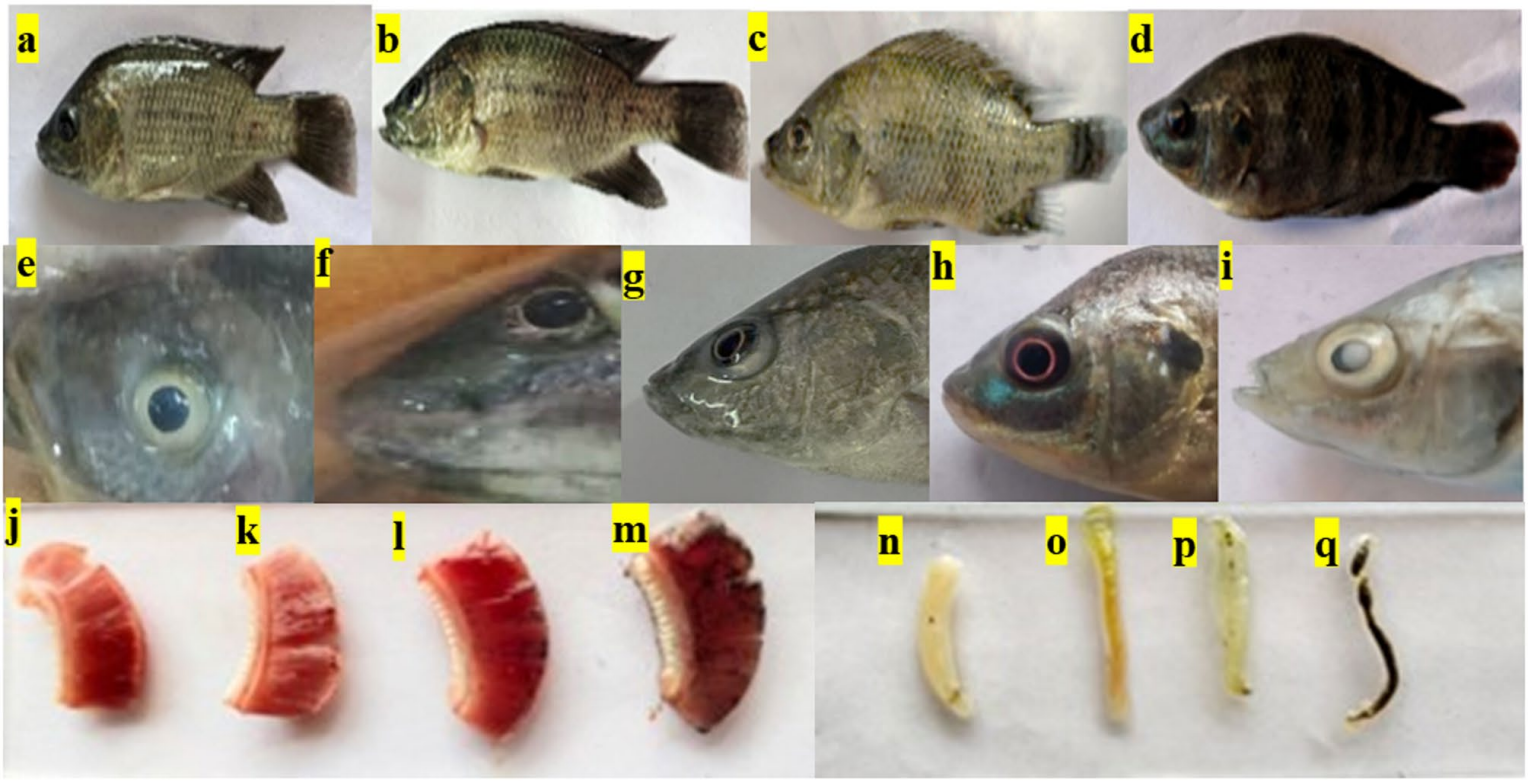
Body color of fish (**a**) Control, (**b**) Drug control, (**c**) Treated water exposed (c) and Dye exposedfish; Eye color of (**e**) Control group fish, (**f**) Drug control goup, (**g**) Treated water exposed group, (**h**) Appearance of red color eye in dye exposed group and (**i**) Appearance of white spot in eye in dye exposed fish at the end of the 96; Gills of fish dissected at the end of 96th h; (**j**) Control, (**k**) Drug control, (**l**)Treated water fish and (**m**) Dye exposed fish; The intestine of fish dissected at the end of 96th h (**n**) Control, (**o**) Drug control, (**p**) Treated water fish and (**q**) Dye exposed fish.

Behavioural and morphological changes in Nile tilapia exposed to dye, compared to control groups, drug control groups, and those exposed to treated water, were analyzed. Fish exposed to the dye exhibited abnormal behaviours, including irregular swimming patterns, rapid opercular movement, and frequent surfacing for air after 48 h. They also showed signs of severe diarrhea initially and lost their appetite after 72 h, likely due to dye accumulation in their digestive tract. In contrast, fish in the control, drug control, and treated water groups maintained normal behaviour throughout the study. Morphological changes were observed in the dye-exposed group after 96 h, including red or white spots on the eyes, skin lesions, and visible dye on their bodies. The control groups maintained clear eyes and consistent coloration (Fig. 5). Mortality was highest in the dye-exposed group, with deaths occurring at 48, 72, and 96 h. The treated water group experienced two deaths, while no mortality was recorded in the control and drug control groups. Dissection revealed dye accumulation in the alimentary canal and gills of the exposed fish, while the control group’s alimentary canal was clear (Fig. 5). The drug control group showed slight changes, and the treated group exhibited a slight yellowish color in the alimentary canal. These findings underscore the significant physiological and behavioural impacts of dye exposure on fish, highlighting the environmental risks of dye pollution in aquatic ecosystems.

### In-vivo histopathological toxicity analysis of BSE- ZnON on *Oreochromis niloticus* (Nile tilapia)

**Fig. 6:**
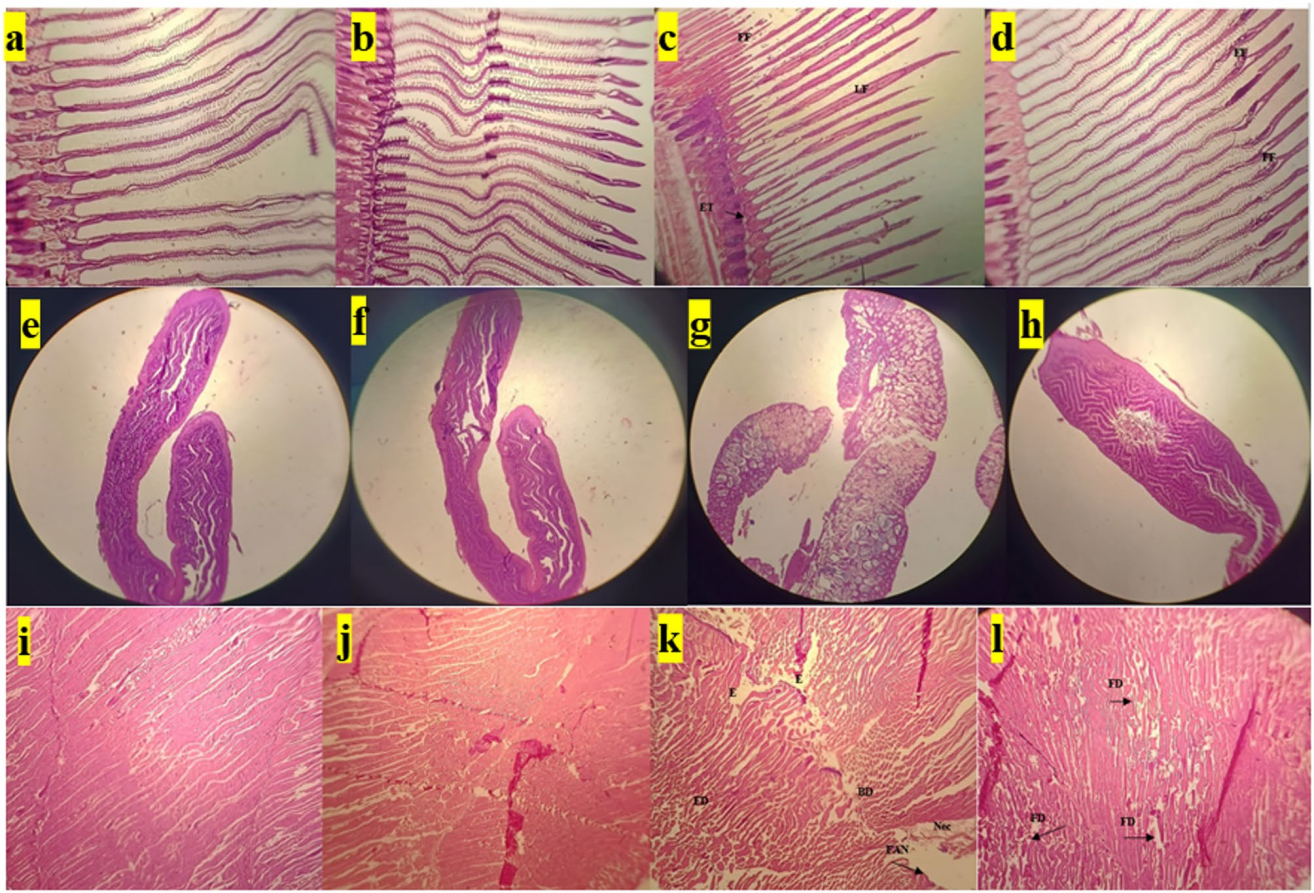
Histopathological alteration of transversely paraffin sectioned muscle of Tilapia electron micrographs. Gill tissue of (**a**) Control, (**b**) drug control, (**c**) dye exposed and (**d**) treated water exposed group; Intestine tissue of (**e**)Control, (**f**) drug control, (**g**) dye exposed and (**h**) treated water exposed group; Muscle tissue of (**i**) Control, (**j**) drug control, (**k**) dye exposed and (**l**) treated water exposed group; *Fd* Fiber damage, *Bd* Bundle damage, *FAN* Focal area of necrosis, *Nec* Necrosis, *E* Edema, *FF* Fractured filament, *LF* Lamellae fusion, *ET* Epithelial thickening, *V* Vacuole and DIS: Disintegration.

Muscle Tissue: Control and drug control groups showed no notable changes, with regular muscle fiber structure and narrow, unstained spaces (Fig. 6i,j). In contrast, dye-exposed fish (Fig. 6k) exhibited severe histopathological alterations, including widened intermuscular connective tissue due to edema, fibrillar and muscle bundle damage, and focal necrosis. The treated water group (Fig. 6l) showed only fibrillar damage, indicating less severe effects compared to the dye-exposed group.

Gill Tissue: Control, drug control, and treated water groups (Fig. 6a–c) displayed normal gill structure with intact secondary lamellae attached to primary lamellae. Dye-exposed gills (Fig. 6d) showed drastic changes, including sloughed-off epithelial cells, fused secondary lamellae, epithelial thickening, and fractured filaments. The treated water group also exhibited some fractured filaments, but the damage was less severe than in the dye-exposed group.

Intestinal Tissue: Control and drug control groups (Fig. 6e–g) maintained normal intestinal structure with an intact mucus layer and healthy microvilli. Dye-exposed intestines (Fig. 6h) displayed severe cellular disintegration, a disrupted mucus layer, and damaged microvilli. The treated water group showed vacuole formation but retained a normal mucus layer, similar to the control group.

These histopathological findings, combined with behavioural and morphological observations, demonstrate the toxic effects of dye exposure on Nile tilapia and the potential of treated water to mitigate these effects. The study highlights the environmental risks of dye pollution and the importance of effective wastewater treatment strategies.

## Discussion

This study synthesised of ZnO nanoparticles using *Biancaea sappan* (BS) leaf extract, marking the first time this particular plant had been utilized for this purpose. *Biancaea sappan* had long been recognized in traditional medicine for its wide array of therapeutic properties, including anticonvulsant, antiproliferative, anticoagulant, anti-inflammatory, antioxidant antiviral, immunostimulant, and antimicrobial effects. The researchers employed a comprehensive suite of characterization techniques, including Field Emission Scanning Electron Microscopy (FESEM), UV-visible spectrophotometry, X-ray diffraction (XRD), Energy Dispersive X-ray spectroscopy (EDX), and Fourier Transform Infrared spectroscopy (FTIR). The results obtained from these analyses aligned closely with earlier reports on ZnO NPs, confirming the successful synthesis of the nanoparticles using BS leaf extract. Previous studies had reported on the remarkable bioactivity of *Biancaea sappan* extract. Notably, it had demonstrated effective DPPH radical scavenging activity, even at low concentrations of 0.98 mg/mL, achieving an impressive 81.67% scavenging rate. Furthermore, BS extract (BSE) exhibited significant anti-inflammatory properties, with 61.9% inhibition of protein denaturation at a concentration as low as 0.1 µg/mL^27,28,39^. The current study successfully replicated these bioactivities, further validating the therapeutic potential of BS.

The researchers attributed the extract’s efficacy to the presence of several bioactive compounds, including hexadecanoic acid, octadecadienoic acid, naphthalene, and linoleic acid. Previous studies had reported on the individual bioactivities of these compounds, supporting the current study’s findings^42,43^. To contextualize their work, the researchers drew comparisons with similar studies in the field. For instance, Sivasankarapillai et al. (2023) synthesized ZnO nanoparticles using *Scoparia dulcis* leaf extract. Their nanoparticles, measuring 20.2 nm and described as pebble-like, exhibited strong antioxidant properties with an IC50 of 1.78 µg/mL in the DPPH assay^25^. Additionally, these nanoparticles showed significant antimicrobial activity against both bacteria and fungi at a concentration of 500 µg/mL. In another relevant study, utilized *Euphorbia hirta* leaf extract for an eco-friendly and efficient synthesis of zinc nanoparticles. Their method was noted for its speed and cost-effectiveness, avoiding the use of hazardous agents^26^. The resulting nanoparticles demonstrated effectiveness against fungi (specifically *Aspergillus niger* and *Cuboida)* and bacteria (*Staphylococcus aureus).*

A key finding of the current study was the synergistic bioactivity observed between *Biancaea sappan* extract and ZnO nanoparticles. Samples treated with BSE-ZnONP exhibited enhanced anti-inflammatory, antioxidant, and antibacterial activities compared to either component alone. This synergistic effect opened up new possibilities for applications in various fields, including medicine and environmental remediation. The researchers also explored the dye degradation capabilities of their BSE-ZnONP composite. They found that their nanoparticles were capable of degrading various toxic dyes, echoing the findings of Priyanka and Naba, who reported that green-synthesized ZnONPs from Hibiscus rosa sinensis could degrade different dyes. Similarly, Vasantharaj. observed efficient photodegradation of toxic dyes using ZnONPs^44^. Although ZnO nanoparticles are known for their photocatalytic activity, in this study, dye degradation occurred under ultrasonication at 20 kHz, which primarily promotes sonocatalysis. The acoustic cavitation effect generates localized high temperatures and pressures, leading to the formation of hydroxyl radicals that facilitate dye degradation. Therefore, the observed enhancement can be attributed to a synergistic sonocatalytic mechanism rather than pure photocatalysis. The current study found their BSE-ZnONP composite to be more efficient in dye degradation compared to previous reports, highlighting its potential for wastewater treatment applications. Similar to the present green synthesis approach, recent research on biomass-derived porous flower-like MoS₂/carbon composites has demonstrated how integrating natural carbon sources can enhance the surface architecture, catalytic activity, and sustainability of nanomaterials. This further supports the environmental relevance and structural advantages of using plant-based materials in functional nanocomposite synthesis. Moreover, the adoption of biomass-derived scaffold/composite structures has been reported recently, for example in MoS₂/carbon composites derived from bagasse, reinforcing the feasibility of integrating waste biomass in advanced functional materials^45^. To optimize the practical application of their nanoparticles, the researchers employed Response Surface Methodology (RSM) to determine the ideal concentration of BSE-ZnONPs. This optimization process not only helped to reduce the quantity of nanoparticles needed for effective treatment but also minimized any potential toxicity of the material.

The safety of the BSE-ZnONP composite was a crucial aspect of the study. In-vivo analysis supported the non-toxic nature of the material, aligning with previous studies by Namvar et al. (2014) that reported on the safety of Biancaea sappan and ZnO nanoparticles in human cells. The researchers also determined the LC50 value, which suggested that the compound was safe for human use. To further investigate the environmental impact and efficacy of their nanoparticles, the researchers conducted studies on Oreochromis niloticus (Nile tilapia), a common freshwater fish species^46^. They examined both the toxicity of the nanoparticles and their ability to degrade dye mixtures in an aquatic environment. The results were striking: fish in untreated, dye-contaminated water showed high mortality rates, while those in water treated with BSE-ZnONPs fared significantly better. The researchers attributed the high mortality in the untreated group to reduced oxygen intake and higher accumulation of dyes in the fish’s intestines and gills. This accumulation not only impaired the proper functioning of gills but also led to decreased oxygen levels in the body and increased uptake of toxic dyes^47^. The current study observed a higher accumulation of dye in the intestines of fish from the untreated group. This finding aligned with research by Mehra et al., (2021) which reported that the accumulation of textile dyes in fish intestines reduced digestion efficiency, weakened the fish, affected normal movement, and caused diarrhea. The researchers in the current study observed similar behavioral changes, confirming that these effects were due to the toxic impact of the dyes^48^. In contrast, fish in the BSE-ZnONP-treated group showed much lower quantities of dye in their intestines and appeared healthier overall. This stark difference underscored the potential of BSE-ZnONPs in mitigating the environmental impact of textile dyes in aquatic ecosystems.

The researchers conducted histopathological analyses to further investigate the impact of dye exposure and treatment. They observed a higher rate of damage to the gills, muscles, and intestines of fish in the untreated group. In comparison, fish treated with BSE-ZnONPs showed significantly less damage to these tissues. These findings were consistent with previous studies, such as that by Suryavathi et al. (2005), which reported that textile dyes could cause cellular damage in the gills, intestines, and muscles of fish^49^.

## Conclusion

The study introduced a novel method for synthesizing ZnO nanoparticles using Biancaea sappan leaf extract and demonstrated the significant potential of these nanoparticles in addressing environmental challenges. The BSE-ZnONP composite exhibited promising capabilities in dye degradation, showing enhanced efficiency compared to previous studies. Furthermore, the study shed important light on the ecological implications of these nanoparticles, emphasizing how they may lessen the negative effects of textile dyes on aquatic life. The optimization of nanoparticle concentration and the confirmation of their safety through in-vivo studies paved the way for practical applications in wastewater treatment and environmental remediation. This research represented a significant step forward in the development of green, efficient, and safe nanotechnological solutions to environmental pollution.

## Data Availability

The datasets used and/or analysed during the current study available from the corresponding author on reasonable request.
